# 5.N. Workshop: Children first: do health systems pay enough attention to their youngest constituents?

**DOI:** 10.1093/eurpub/ckac129.330

**Published:** 2022-10-25

**Authors:** Organised by: European Observatory on Health Systems and Policies, Chair persons: Ewout Van Ginneken (Germany), Dimitra Panteli (Belgium)

## Abstract

This workshop aims to bring together a range of insights on how health systems have dealt with child health and health care, highlighting both successes and challenges. For children, access to health care is vital. It impacts their physical and emotional health and wellbeing and largely determines whether they can reach their full potential as adults, which is also important to their families and society at large. Consequently, it comes as no surprise that he 1989 UN Convention on the Rights of the Child, which was ratified by all European countries, guarantees a fundamental right to healthcare for all children. However, various national governments do not fully comply with the Convention, and evidence exists that children may not always enjoy the necessary access to meet their needs. In 2021, Doctors of the World (DoW) concluded that there are urgent questions about the welfare and health of vulnerable children across Europe, based on the increase in children seeking healthcare from DoW clinics instead of the national health system. This concerning trend is not captured well enough in available statistics. What is more, the COVID-19 pandemic and the Ukrainian refugee crisis raise additional concerns as to how health systems will deal with the observed increases in related paediatric sequelae and mental health needs. The aims of this workshop are therefore to stimulate discussion around better understanding children's access to health care and how it relates to health system performance, and provide impetus for new initiatives that can improve health care for children. The workshop will take a hybrid format, kicking off with three short presentations followed by a round table discussion and engagement with the audience. The presentations will cover the regulatory framework for children's access to health care in European countries; identified bottlenecks of health system performance specific to child services from a range of countries around the world; and the impact of COVID-19 on hospital care for children based on the example of one European country.

**Key messages:**

• Ensuring children’s access to health care is now more important than ever; a lot more can and should be done at all levels.

• More and better data about children’s health and health care access is necessary to enable effective policies; collecting it should become a health system priority.

